# Beyond attenuation: a translational review of curative-intent pharmacological targets in idiopathic pulmonary fibrosis

**DOI:** 10.3389/fmed.2026.1748453

**Published:** 2026-04-21

**Authors:** Ghaith K. Mansour, Ahmad W. Hajjar, Hatouf H. Sukkarieh

**Affiliations:** 1 Alfaisal University, Riyadh, Saudi Arabia; 2 College of Medicine, Alfaisal University, Riyadh, Saudi Arabia; 3 College of Medicine, Department of Pharmacology, Alfaisal University, Riyadh, Saudi Arabia

**Keywords:** autotaxin, clinical trials, idiopathic pulmonary fibrosis, integrins, novel pharmacological approaches, senolytics, translational medicine

## Abstract

Idiopathic Pulmonary Fibrosis (IPF) is a progressive, fatal lung disease with a median survival of 3–5 years, driven by a profound unmet medical need. The current standard-of-care agents, pirfenidone and nintedanib, merely slow disease progression and are burdened by significant toxicity. This review synthesizes the basic, clinical, and translational evolution of novel pharmacological approaches for IPF, analyzing the critical lessons from recent high-profile clinical trial failures and successes. This review followed a narrative, analytical methodology, examining key Phase 2 and 3 clinical trials to identify a “failure-to-refinement” trajectory in drug development. Analysis reveals that targeting broad-spectrum enzymes (e.g., autotaxin via ziritaxestat) or downstream effectors (e.g., Connective Tissue Growth Factor (CTGF) via pamrevlumab) has failed, likely due to mechanistic redundancy or insufficient target engagement. In contrast, highly specific, next-generation inhibitors targeting upstream signal initiation points such as the lysophosphatidic acid receptor 1 (LPAR1) (admilparant) and local αv*β*6 integrin-mediated activation of transforming growth factor-beta (TGF-β) (bexotegrast) have yielded promising Phase 2 data, demonstrating a sophisticated translational learning loop. Furthermore, the geroscience approach targeting cellular senescence (dasatinib/quercetin) has introduced a controversial new paradigm, while the recent approval of nerandomilast (a phosphodiesterase 4B (PDE4B) inhibitor) establishes a new therapeutic class. The IPF pipeline has matured, moving from broad attenuation toward specific, mechanism-based inhibition. This shift, informed by interpreting trial failures, is guiding the development of therapies toward disease modification and combination strategies, offering a tangible path toward curative-intent treatments.

## 1 Introduction

IPF is defined as a specific form of chronic, progressive fibrosing interstitial pneumonia of unknown cause (1). This devastating disease occurs primarily in older adults and is limited to the lungs, distinct from other interstitial lung diseases (2). The diagnosis requires the rigorous exclusion of other known causes of interstitial lung diseases, such as environmental exposures, connective tissue disease, or drug toxicity (1). Diagnosis is confirmed by the presence of a Usual Interstitial Pneumonia (UIP) pattern on high-resolution computed tomography (3) without tissue confirmation in suitable clinical settings, especially when backed by multidisciplinary dialogue. Not every patient needs a surgical lung biopsy (1). The evolution of these diagnostic criteria from the initial (4) consensus statement to the 2018 multidisciplinary guidelines has been essential for standardizing clinical trial populations (1). Despite diagnostic refinement, the prognosis remains dismal, with a median survival of only 3–5 years from diagnosis (5).

The current pharmacological standard of care (SoC) for IPF consists of two conditionally recommended antifibrotic agents, pirfenidone and nintedanib (6, 7). The approval of these agents was based on landmark Phase 3 trials: ASCEND for pirfenidone, which demonstrated a reduction in disease progression (8), and the INPULSIS-1 and INPULSIS-2 trials for nintedanib, which showed a significant reduction in the annual rate of decline in forced vital capacity (FVC) (9). Pirfenidone’s precise mechanism is not fully elucidated, but it is known to inhibit fibroblast proliferation and collagen synthesis by interfering with TGF-*β* signaling (10). Preclinical data demonstrate that pirfenidone specifically impacts the TGF-β–mediated phosphorylation of Smad3, a key downstream mediator of its antifibrotic effect (11). Nintedanib is an intracellular, small-molecule tyrosine kinase inhibitor (12). It functions by competitively blocking the adenosine triphosphate binding pocket of multiple pro-fibrotic receptors, chiefly platelet-derived growth factor receptor, fibroblast growth factor receptor, and vascular endothelial growth factor receptor (13). The mechanisms of action, primary efficacy, and key adverse events (AE) of the current standard-of-care antifibrotics, pirfenidone and nintedanib, are summarized in Table 1, highlighting their pharmacological profiles and clinical limitations.

**Table 1:** Mechanisms, efficacy, and key adverse events of current standard-of-care antifibrotics.

Drug	Target/mechanism of action	Primary efficacy/endpoint	Key adverse events and contraindications and limitations	Relevant citations
Pirfenidone	Possesses anti-fibrotic, anti-inflammatory, and antioxidant properties. Inhibits the TGF-β/SMAD3 signaling pathway.	Slows the rate of FVC decline by attenuating extracellar matrix accumulation. Validated by the ASCEND trial, which its primary endpoint was significant reduction in the proportion of patients with a decline in FVC or death over 52 weeks	Gastrointestinal: Nausea, dyspepsia, anorexia. Dermatologic: Photosensitivity, rash. Contraindicated in severe hepatic or renal impairment Limitations: Efficacy is reduced by cigarette smoking (CYP1A2 induction).	(8, 16, 74–80)
Nintedanib	Small-molecule tyrosine kinase inhibitor. Blocks receptors for platelet-derived growth-receptor, fibroblast growth factor receptor, and vascular endothelial growth factor receptor.	Slows the rate of FVC decline by inhibiting fibroblast proliferation, migration, and differentiation. Validated by the INPULSIS-1 and INPULSIS-2 trials, which their primary endpoints by significantly reducing the annual rate of decline in FVC (mL/year) over 52 weeks.	Gastrointestinal: Diarrhea (most common, >58%), nausea, vomiting. Hepatic: Elevated liver enzymes. Contraindications: 1-pregnancy (Teratogenic). 2-severe hepatic impairment. Limitations: Use with caution in patients with bleeding risk or those on full-dose anticoagulation (vascular endothelial growth factor receptor inhibition)	(9, 13, 16, 74, 78, 79, 81, 82, 84)

Both therapies, however, are fundamentally limited, as they only slow the rate of decline and do not halt disease progression (14) or reverse established fibrosis (15). Their utility is further compromised by significant AE profiles that challenge patient adherence and quality of life (6). Nintedanib is associated with high rates of gastrointestinal toxicity, with over 58% of patients experiencing diarrhea (16). Pirfenidone is primarily associated with photosensitivity and gastrointestinal discomforts such as nausea and anorexia (16, 17). These limitations create a profound and urgent unmet medical need for novel pharmacological approaches that offer superior efficacy and improved tolerability (14, 18, 19). This review analyzes the translational trajectory of emerging IPF therapies, focusing on a “failure-to-refinement” narrative that is guiding the field toward mechanism-specific, curative-intent strategies.

## 2 Targeting cellular senescence: the geroscience approach

### 2.1 Mechanistic rationale: the senescent alveolar epithelium

IPF is now widely recognized as a disease of premature and progressive cellular senescence (CS), linking its pathogenesis directly to the biology of aging (20–22). This paradigm is supported by the accumulation of senescent alveolar epithelial cells and lung fibroblasts in IPF lung tissue (23). One of the primary drivers of this premature senescence is telomere attrition, a key hallmark of aging (24, 25). Germline mutations in telomerase genes, such as telomerase reverse transcriptase, which are responsible for telomere maintenance, represent the most significant genetic risk factor for familial pulmonary fibrosis and are also found in sporadic IPF (26). This telomere dysfunction triggers a DNA damage response in alveolar epithelial cells, leading to replicative senescence and an inability to conduct normal epithelial repair following repetitive micro-injury (27).

Senescent cells exit the cell cycle and develop a pro-inflammatory, pro-fibrotic senescence-associated secretory phenotype (28). The senescence-associated secretory phenotype is a complex secretome of cytokines (e.g., IL-6, IL-8), growth factors (e.g., TGF-*β*), and matrix-remodeling enzymes (22, 23). This toxic microenvironment acts in a paracrine fashion to promote the proliferation and differentiation of underlying fibroblasts into activated myofibroblasts, which are the primary effectors of extracellular matrix deposition (23, 29). Preclinical validation of this pathway demonstrated that senescent cells secrete functional leukotrienes, which contribute significantly to the fibrotic milieu (30). Furthermore, the selective depletion of senescent epithelial cells *in vitro* and ex vivo was shown to stabilize the epithelial phenotype and decrease fibrotic markers (31). Targeted elimination of p16Ink4a-positive fibroblasts using senolytic compounds reduced fibrotic burden in mouse models and in precision-cut human IPF lung slices (32, 33).

### 2.2 Clinical translation and controversy

The geroscience hypothesis was tested in a landmark first-in-human, open-label pilot study (NCT02874989) utilizing the senolytic combination of dasatinib and quercetin (D + Q) in 14 patients with stable IPF (34, 35). The trial successfully met its primary endpoints of feasibility and safety, demonstrating that an intermittent senolytic dosing regimen was well-tolerated over a 3-week period (34). The study generated profound interest, however, from its secondary endpoints, which revealed a statistically significant and clinically meaningful improvement in physical function, including 6-min walk distance (6MWD), 4-meter gait speed, and chair-stand time (34, 36). This functional improvement occurred in the absence of any corresponding change in pulmonary function tests, frailty indices, or patient-reported quality of life (34).

This striking disconnect improved physical function without improved lung function—suggests the therapeutic effect may be systemic rather than pulmonary (21). Given that senescence is a fundamental driver of systemic frailty, D + Q may have improved musculoskeletal function and reduced systemic inflammation, thereby treating the patient’s aging rather than the lung’s fibrosis (22). This optimism was quickly tempered by significant safety concerns that have emerged from subsequent trials (24). A Phase 1 randomized controlled trial reported that the D + Q group experienced nearly three times more acute exacerbations than the placebo group, alongside significant general and gastrointestinal discomfort (35). This has created a critical research gap, leaving the senolytic approach as a high-risk, high-reward strategy that requires substantial further investigation to determine if it can be safely harnessed for IPF (24, 37).

## 3 The autotaxin-LPA-LPAR1 axis: a translational “failure-to-refinement” narrative

### 3.1 Mechanistic rationale: the LPA-LPAR1 signal

The autotaxin–lysophosphatidic acid (LPA) signaling axis has been identified as a critical, druggable pathway in the pathogenesis of pulmonary fibrosis (38, 39). ATX (ENPP2) is a secreted lysophospholipase D, and it is the principal enzyme responsible for the extracellular conversion of lysophosphatidylcholine to the bioactive lipid LPA (40). LPA mediates a multitude of pro-fibrotic processes by signaling through at least six G-protein coupled receptors, with the LPA1 receptor (LPAR1) being the key effector in lung fibrosis (38, 41). Activation of LPAR1 on fibroblasts promotes cell migration, proliferation, survival, and αv*β*6-mediated activation of TGF-β, driving the fibrotic cascade (41, 42). Genetic deletion of LPAR1 in mice provided powerful preclinical validation, demonstrating significant protection from bleomycin-induced pulmonary fibrosis (43).

### 3.2 Clinical failures and translational insights

This strong preclinical rationale led to the development of ziritaxestat (GLPG1690), a small-molecule inhibitor of the enzyme ATX (44). Following a promising Phase 2a trial (FLORA), ziritaxestat was advanced into two large, identically designed Phase 3 trials, ISABELA 1 (NCT03711162) and ISABELA 2 (NCT03733444) (45–47). In 2021, the entire ISABELA program was halted by the independent data monitoring committee (46). The trials were terminated due to a lack of efficacy, as ziritaxestat failed to demonstrate any reduction in the 52-week rate of FVC decline compared to placebo (46, 48). Furthermore, pooled data showed a concerning, numerically higher rate of all-cause mortality in the ziritaxestat treatment groups (46).

Concurrently, a different therapeutic strategy targeting the receptor LPAR1, BMS-986020, was evaluated in a phase 2 trial (NCT01766817) (49). This trial produced the opposite result: treatment with BMS-986020 600 mg twice daily did significantly slow the rate of FVC decline over 26 weeks compared to placebo (*p* = 0.049) (49). Despite this efficacy, this trial was also terminated early, but for reasons of off-target toxicity, specifically dose-related elevations in hepatic enzymes and three cases of drug-related cholecystitis (49, 50).

### 3.3 Next, steps: a refined target

The concurrent failures of ziritaxestat (ineffective) and BMS-986020 (toxic) provided an invaluable, albeit costly, translational lesson (38). The failure of the ATX inhibitor ziritaxestat may be attributable to suboptimal target engagement or the presence of redundant LPA production pathways (38, 40). Conversely, the efficacy of BMS-986020 demonstrated that LPAR1 itself is a valid, high-potential therapeutic target in human IPF and that the drug’s formulation, not the mechanism, was the problem (38, 49). This rational interpretation guided the development of admilparant (BMS-986278), a next-generation, highly selective LPAR1 antagonist designed to retain efficacy while eliminating the off-target hepatotoxicity (51).

A recent Phase 2 trial (NCT04308681) evaluating admilparant in parallel IPF and PPF cohorts reported encouraging results (51). In the IPF cohort, the 60 mg twice-daily dose of admilparant resulted in a 62% relative reduction in the rate of decline in percent predicted FVC versus placebo over 26 weeks (52). Critically, admilparant was safe and well-tolerated, with rates of AEs comparable to placebo and no evidence of the hepatobiliary toxicity that halted its predecessor (51). These data strongly support the progression of admilparant into Phase 3 trials, representing a clear success of the translational “failure-to-refinement” cycle (38, 51).

However, these findings must be interpreted with caution given the limitations inherent to Phase 2 designs, including smaller sample sizes and relatively short treatment durations (26 weeks) to assess disease progression (53).

## 4 Targeting pro-fibrotic mediators: from broad failure to specific success

### 4.1 The TGF-*β* and CTGF conundrum

TGF-β is universally recognized as the master pro-fibrotic cytokine and a central mediator of fibrosis (54, 55). However, its functional pleiotropy, including essential homeostatic roles in immune regulation, cell proliferation, and normal tissue repair, makes systemic TGF-β inhibition a highly controversial and potentially dangerous strategy (56, 57). Direct blockade carries a high risk of adverse effects, such as uncontrolled inflammation or impaired wound healing (57). This challenge led researchers to target key downstream mediators of TGF-β’s pro-fibrotic activity, most notably CTGF (58).

Pamrevlumab, a fully human monoclonal antibody against CTGF, was developed to disrupt this downstream signaling (59). After the Phase 2 PRAISE trial (NCT01890265) showed a promising reduction in FVC decline, pamrevlumab was advanced to a large Phase 3 program (60, 61). The Phase 3 ZEPHYRUS-1 trial (NCT03955146) was a definitive failure, prompting the discontinuation of the entire program (62). The study found no statistically significant difference in the primary endpoint of absolute change in FVC from baseline to week 48 (*p* = 0.29) between the pamrevlumab and placebo groups (62).

### 4.2 A refined mechanistic approach: integrin inhibition

The failure of downstream CTGF inhibition, analogous to the failure of ATX inhibition, suggests that the TGF-β pathway likely acts through multiple redundant effectors, rendering the blockade of a single one insufficient (57). This translational insight, combined with the dangers of systemic TGF-β blockade, has refocused development on a more sophisticated strategy: inhibiting the local activation of latent TGF-β at the precise site of lung injury (63). In the lung, latent TGF-β is tethered to the extracellular matrix until it is activated by mechanical force applied by the αvβ6 integrin, which is highly upregulated on injured alveolar epithelial cells (64, 65). This localized activation is the initiating step of the fibrotic cascade (63, 66). Preclinical validation was definitive: mice genetically engineered to lack the β6 integrin subunit are completely protected from pulmonary fibrosis (65).

### 4.3 Clinical translation

This mechanism is the target of bexotegrast (PLN-74809), an oral, once-daily, dual-selective inhibitor of the αvβ6 and αvβ1 integrins (67). The 12-week, Phase 2a INTEGRIS-IPF trial (NCT04396756) randomized IPF patients (with or without background SoC) to various doses of bexotegrast or placebo (67). The trial successfully met its primary endpoint, demonstrating a favorable safety and tolerability profile (67). On exploratory efficacy endpoints, bexotegrast suggested a dose-dependent reduction in FVC decline versus placebo (67). These positive data support the advancement of this mechanism-specific strategy, reinforcing the paradigm that targeting local, upstream activators is a more viable approach than blocking either systemic sources or downstream effectors (67).

## 5 The new wave of approved and emerging targets

A major development in the IPF pipeline was the recent success of the Phase 3 FIBRONEER-IPF trial (NCT05321069). This study evaluated nerandomilast (BI 1015550), a first-in-class, preferential phosphodiesterase 4B (PDE4B) inhibitor (68). The PDE4B enzyme is implicated in (68) both pro-fibrotic and pro-inflammatory pathways, and its inhibition offers a novel dual-action mechanism (68). The trial met its primary endpoint, showing a significantly smaller FVC decline in the nerandomilast group compared to placebo (68), and subsequently received U. S. Food and Drug Administration (FDA) approval in late 2024 (69). A translational summary of novel pharmacological targets and drug candidates in the IPF pipeline, detailing their mechanisms, clinical trial status, and key findings, is presented in Table 2.

**Table 2:** Translational summary of novel pharmacological targets for idiopathic pulmonary fibrosis.

Pharmacological target	Drug candidate	Mechanism of action	Key clinical trial(s)	Phase	Status/key finding (efficacy and safety)	Relevant citations
Cellular Senescence	Dasatinib + Quercetin (D + Q)	Senolytic: Induces apoptosis of senescent cells.	Open-label Pilot (NCT02874989)	Phase 1/Pilot	Efficacy: Improved physical function (6MWD). Safety: Feasible, but subsequent trials report increased AEs and exacerbations.	(34, 35)
Autotaxin (ATX)	Ziritaxestat (GLPG1690)	Inhibitor of ATX enzyme; blocks LPA production.	ISABELA 1 and 2 (NCT03711162)	Phase 3	Terminated: Failed to meet primary FVC endpoint; numerically higher mortality signal.	(46)
LPAR1	BMS-986020	LPAR1 antagonist; blocks LPA signaling.	(NCT01766817)	Phase 2	Terminated: Efficacious (slowed FVC decline) but terminated for off-target hepatobiliary toxicity.	(49)
LPAR1	Admilparant (BMS-986278)	Next-generation selective LPAR1 antagonist.	(NCT04308681)	Phase 2	Positive: Slowed FVC decline (62% relative reduction); well-tolerated with no liver toxicity signal.	(82)
CTGF	Pamrevlumab	Monoclonal antibody; neutralizes CTGF.	ZEPHYRUS-1 (NCT03955146)	Phase 3	Terminated: Failed to meet primary FVC endpoint (no significant difference vs. placebo).	(59)
αvβ6 Integrin	Bexotegrast (PLN-74809)	Oral inhibitor of αvβ6; blocks local TGF-β activation	INTEGRIS-IPF (NCT04396756)	Phase 2a	Positive: Favorable safety profile; demonstrated dose-dependent slowing of FVC decline.	(64)
Phosphodiesterase 4B (PDE4B)	Nerandomilast	Preferential PDE4B inhibitor; antifibrotic and immunomodulatory.	FIBRONEER-IPF (NCT05321069)	Phase 3	Positive: Met primary endpoint (slowed FVC decline vs. placebo); FDA Approved.	(83)

The pipeline beyond this remains robust, with over 40 agents in Phase 2 or higher clinical trials. Other novel mechanisms under investigation include ifetroban, a thromboxane-prostanoid receptor (TBXA2R) antagonist that recently entered a Phase 2 trial (NCT05571059). This target is based on preclinical models linking oxidative stress to fibroblast activation during lung fibrosis. Other novel agents currently under investigation include the hedgehog pathway inhibitor Taladegib (70), the ROCK2 inhibitor Zelasudil (70), and the angiotensin II type 2 receptor agonist Buloxibutid (71). Despite this progress, fundamental research gaps in IPF pathogenesis remain, particularly regarding the initial trigger for epithelial injury and the precise mechanisms preventing normal alveolar repair (72). Furthermore, a systems medicine approach is increasingly necessary as the IPF population ages (5). Patients with IPF suffer from extensive multimorbidity, and the effective management of common comorbidities, such as gastro-esophageal reflux disease (GORD), ischemic heart disease, and hypertension, is a critical and integrated component of optimal IPF care (7, 73).

## 6 Discussion

The pharmacological treatment of IPF is undergoing a critical maturation, driven by a translational “failure-to-refinement” narrative (4). For a decade, the field was defined by the modest efficacy and high toxicity of pirfenidone and nintedanib (6). The subsequent pursuit of novel agents led to high-profile Phase 3 failures, including the ATX inhibitor ziritaxestat and the CTGF inhibitor pamrevlumab (46, 62). These failures were not setbacks but rather essential translational lessons, suggesting that targeting broad enzymes or downstream effectors is an insufficient strategy, likely due to mechanistic redundancy (38, 57), though issues regarding trial design, patient selection, and sufficient target engagement cannot be fully ruled out.

This has guided the successful development of next-generation, highly specific molecules that target the “sweet spot” of signal initiation (38). The promising Phase 2 data from the LPAR1 antagonist admilparant and the αv*β*6 integrin inhibitor bexotegrast support this refined strategy of targeting specific upstream receptors and local activators (51, 67). This approach maximizes mechanistic specificity at the site of injury while minimizing the risk of systemic, off-target toxicity (51). Simultaneously, the geroscience paradigm has opened a new, controversial front (20). The pilot data from D + Q, showing improved physical but not pulmonary function, forces a critical re-evaluation of endpoints and safety, questioning whether we are treating systemic frailty or pulmonary fibrosis (34, 35). The recent approval of the PDE4B inhibitor nerandomilast provides a validated new therapeutic class, further expanding the armamentarium (69).

## 7 Conclusion and future directions

The pharmacological landscape of IPF is at a pivotal inflection point. For the first time, the field is moving beyond the limited efficacy and significant toxicity of the original standard-of-care agents. The recent failures of broad-target drugs have provided invaluable lessons, steering development toward highly specific, mechanistically precise molecules that target critical nodes of fibrotic activation, such as LPAR1 and αv*β*6 integrin. The recent approval of nerandomilast, the first new drug in a decade, validates a new mechanism and provides a much-needed additional tool.

Future research must now focus on three key areas: (1) Translating the most promising Phase 2 agents, like admilparant and bexotegrast, into Phase 3 successes; (2) Resolving the critical safety and efficacy questions surrounding geroscience-based approaches like senolytics; and (3) Designing intelligent clinical trials to test rational, multi-agent combination therapies. The ultimate goal is to shift the treatment paradigm from merely slowing FVC decline to actively halting fibrosis and promoting lung repair, finally offering a curative-intent strategy for this devastating disease.
